# Fluidic shaping and in-situ measurement of liquid lenses in microgravity

**DOI:** 10.1038/s41526-023-00309-9

**Published:** 2023-09-11

**Authors:** Omer Luria, Mor Elgarisi, Valeri Frumkin, Alexey Razin, Jonathan Ericson, Khaled Gommed, Daniel Widerker, Israel Gabay, Ruslan Belikov, Jay Bookbinder, Edward Balaban, Moran Bercovici

**Affiliations:** 1 Faculty of Mechanical Engineering, Technion – Israel Institute of Technology, Haifa, Israel; 2 NASA Ames Research Center, Moffett Blvd., Moffett Field, CA USA; 3 Present Address: Department of Mathematics, Massachusetts Institute of Technology, Cambridge, MA USA

**Keywords:** Mechanical engineering, Applied optics, Fluid dynamics

## Abstract

In the absence of gravity, surface tension dominates over the behavior of liquids. While this often poses a challenge in adapting Earth-based technologies to space, it can also provide an opportunity for novel technologies that utilize its advantages. In particular, surface tension drives a liquid body to a constant-mean-curvature shape with extremely smooth surfaces, properties which are highly beneficial for optical components. We here present the design, implementation and analysis of parabolic flight experiments demonstrating the creation and in-situ measurement of optical lenses made entirely by shaping liquids in microgravity. We provide details of the two experimental systems designed to inject the precise amount of liquid within the short microgravity timeframe provided in a parabolic flight, while also measuring the resulting lens’ characteristics in real-time using both resolution target-imaging and Shack-Hartmann wavefront sensing. We successfully created more than 20 liquid lenses during the flights. We also present video recordings of the process, from the lenses’ creation during microgravity and up until their collapse upon return to gravity. The work thus demonstrates the feasibility of creating and utilizing liquid-based optics in space.

## Introduction

Optical components are used in a wide range of space applications, including imaging, spectroscopy, communications, and solar concentration^1–3^. Fabrication of optical components is traditionally based on mechanical processes such as grinding and polishing that produce significant waste and rely on heavy machinery^4–8^. Such processes are therefore not suitable for implementation in space. Although 3D printing overcomes many of these limitations and has been successfully utilized in space, it cannot yet produce optical-grade components due to the resulting low surface quality^9–11^. As a result, currently all optical components, from small lenses to large-scale telescope mirrors, are fabricated entirely on Earth and launched into space^5,12^. The ability to manufacture optical components in space could greatly benefit long-duration missions (e.g. to Mars) that must be self-sufficient, as well as open the door to creation of large-scale telescopes, breaking away from the current limitations imposed by the launch process^13–15^.

The use of liquids as a method for creating space optics has been of interest for decades. One prominent example is the ‘spinning liquid telescope’ which subjects reflective liquids to centrifugal forces to create a parabolic mirror. Such telescopes have been demonstrated on Earth^16–18^ and have been suggested as a technology for a lunar-based telescope^19^. However, this approach is not suitable for use in microgravity conditions because, in addition to spinning, it also requires a uniform body force along the optical axis to form the parabolic shape. Moreover, implementation of such telescopes requires high mechanical precision, dynamic stability, and continuous energy consumption to sustain their shapes^17^. Another use of liquids in optics are the so called ‘liquid lenses’, wherein an optical liquid is confined between two elastic membranes, and the change in focal length is achieved by mechanical or electrical actuation of the liquid^20–22^. These ‘liquid lenses’ provide rapid changes in focal length, making them attractive for machine vision applications^23^. Through parabolic flight experiments, Newman and Stephens tested the effect of microgravity on their performance^24^. While this technology is likely useful in space, it still relies on high-quality solid components (e.g. the membrane) that must be prepared on earth, limiting its use and scale.

Recently, a new method for additive manufacturing of optical components was reported^25^. The method, termed ‘Fluidic Shaping’, uses surface tension under neutral (or near neutral) buoyancy conditions to shape a volume of liquid into useful optical components by contacting it with a rigid bounding frame that serves as a spatial constraint. Neutral buoyancy is achieved using an insoluble immersion liquid that has equal density to that of the optical liquid. Both spherical and aspherical lenses, as well as freeform components, can be produced, with the specific topography controlled by the shape of the bounding frame and the level of deviation from neutral buoyancy^26^. In perfect neutral buoyancy, surface tension dominates completely and results in scale-invariant constant-mean-curvature surfaces. For a circular flat boundary (a simple ring) the liquid volume takes a spherical cap shape^25^, with a curvature (positive or negative) determined by the volume of the optical liquid. The component can either remain liquid and thus allow for dynamic control of its curvature by adding or aspirating liquid, or be solidified (e.g. polymerized) to form a solid object.

Owing to its simplicity and inherent compatibility with microgravity, Fluidic Shaping has the potential to serve as a method for in-space manufacturing of high-quality optical components. Moreover, microgravity eliminates the need for a matching immersion liquid. This further simplifies the process relative to its Earth-based implementation by making it independent of the liquid’s absolute density or solubility. Due to the scale invariance of the method under perfect microgravity conditions, optical components of any size can be theoretically produced, while maintaining the same surface quality.

In this work we present the use of Fluidic Shaping in the creation of liquid optical lenses in microgravity without an immersion liquid, in a series of parabolic flight tests. We show the feasibility of the method by injecting the lens liquid into a bounding frame during microgravity, resulting in 20 successful deployments of liquid lenses suspended in air. We present the performance of the resulting lenses obtained by in-situ resolution-target imaging and Shack-Hartmann wavefront sensing, within the short microgravity time frame. The results are accompanied by a detailed design of the hardware, a discussion of design considerations, and guidelines for the construction of such setups.

While our demonstration here was limited to a relatively small scale—due to practical constraints of the in-flight experimental environment—it clearly demonstrates the feasibility of creating liquid optics in microgravity. This potentially opens the door to in-space fabrication of optical components and provides a path toward space telescopes that are based on deployment of liquids in space, on scales that cannot be reached using today’s technologies.

## Methods

### Scientific background

**Fig. 1:**
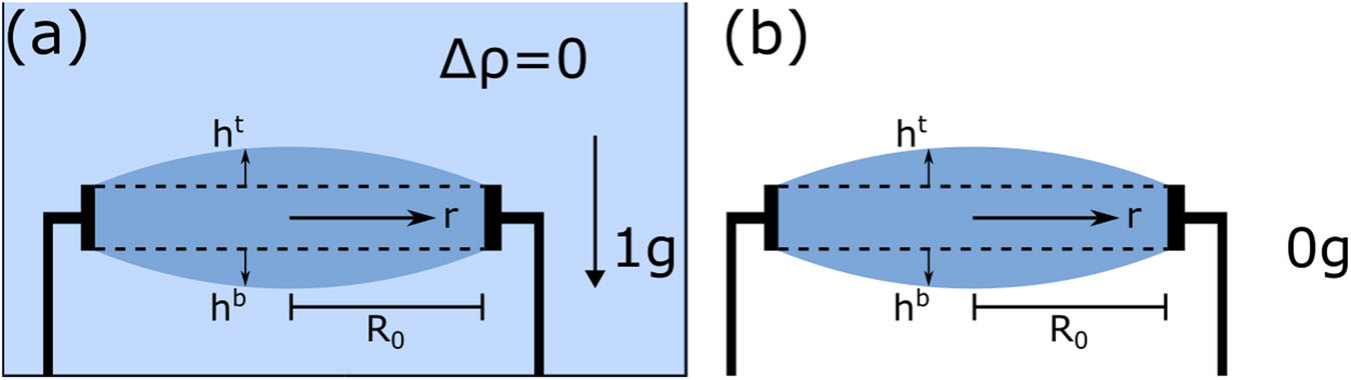
The principle of fluidic shaping, with and without an immersion liquid. When body forces are negligible and surface tension dominates, an optical liquid injected into a circular frame will form a symmetric lens composed of two spherical caps whose curvature is dictated by the volume of the liquid. **a** In the lab, gravitational forces are balanced by injecting the liquid into an immersion liquid of equal density, creating neutral buoyancy conditions. **b** In microgravity, the same result should be obtained without the need for an immersion liquid.

The physical principles of Fluidic Shaping under buoyancy conditions are presented in detail in Frumkin et al. and in Elgarisi et al.^25,26^. For completeness, we here provide a brief overview of these principles, with a focus on microgravity conditions. Consider a volume of optical liquid of density *ρ* that is injected into a circular boundary of radius ${R}_{0}$ and vertical thickness *d*, submerged within another immiscible fluid of density ${\rho }_{{im}}$ (liquid or gas, called here the ’immersion fluid’), as shown in Fig. 1a. Assuming that the optical liquid is pinned on the edges of the boundary, assuming axis-symmetry, and denoting the top and bottom liquid interfaces as ${h}^{t}$ and ${h}^{b}$, the energy of the system can be expressed as

1 $$\begin{array}{lll}E&=&2{\rm{\pi }}\gamma {\displaystyle{\int }}_{0}^{{R}_{0}}\Bigg[\sqrt{1+{\left({h}_{r}^{(t)}\right)}^{2}}+\sqrt{1+{\left({h}_{r}^{(b)}\right)}^{2}}\\ &&-\, \frac{\varDelta \rho g}{2\gamma }\left({\left({h}^{(t)}\right)}^{2}-{\left({h}^{(b)}\right)}^{2}\right)+\frac{\lambda }{\gamma }\left({h}^{(t)}+{h}^{(b)}\right)\Bigg]{rdr}\end{array}$$

Under the volumetric constraint,

2 $${V}_{{lens}}={\rm{\pi }}{R}_{0}^{2}d+2\pi {\int }_{0}^{{R}_{0}}\left({h}^{(t)}+{h}^{(b)}\right){rdr}$$

where the first term in the integral represents the surface energy of the liquid-fluid interfaces, the second represents the gravitational potential energy that includes Earth’s gravity and the hydrostatic buoyancy force, and the third represents a constraint on the finite volume of the injected liquid. *r* and *θ* are the radial and azimuthal coordinates, *γ* is the interfacial energy between the two liquids, $g$ is Earth’s gravity, and *λ* is a Lagrange multiplier. The minimum energy state, where Π has a global minimum, can be expressed by the Euler-Lagrange equations:

3 $$\begin{array}{l}\left(\frac{\Delta \rho g}{\gamma }{h}^{t}-\frac{\lambda }{\gamma }\right)+\frac{r{h}_{{rr}}^{t}+{h}_{r}^{t}+{\left({h}_{r}^{t}\right)}^{3}}{r{\left(1+{\left({h}_{r}^{t}\right)}^{2}\right)}^{\frac{3}{2}}}=0 \\ \left(-\frac{\Delta \rho g}{\gamma }{h}^{b}-\frac{\lambda }{\gamma }\right)+\,\frac{r{h}_{{rr}}^{b}+{h}_{r}^{b}+{\left({h}_{r}^{b}\right)}^{3}}{r{\left(1+{\left({h}_{r}^{b}\right)}^{2}\right)}^{\frac{3}{2}}}=0\end{array}$$

For the case of perfect neutral buoyancy ($\Delta \rho =0$, Fig. 1a), or under microgravity conditions ($g=0$, Fig. 1b) the potential energy term vanishes, the system becomes symmetric (${h}^{t}={h}^{b}$), and the shapes of each of the interfaces can be described by

$$\frac{r{h}_{{rr}}+{h}_{r}+{\left({h}_{r}\right)}^{3}}{r{\left(1+{({h}_{r})}^{2}\right)}^{3/2}}=\frac{\lambda }{\gamma }$$

The left-hand-side of the expression represents the mean curvature of the surface, which as indicated by the right-hand-side, is constant and determined by the volume of the injected optical liquid. For a circular boundary, the shape of the interface is a spherical cap, thus forming a symmetric (bi-convex or bi-concave) spherical lens with a paraxial focal length of

4 $$f={\left[\left(n-1\right)\left(2\frac{\lambda }{\gamma }+{\left(\frac{\lambda }{\gamma }\right)}^{2}\frac{\left(n-1\right)\left(d+2{h}_{r=0}\right)}{n}\right)\right]}^{-1}$$

### Reporting summary

Further information on research design is available in the Nature Research Reporting Summary linked to this article.

## Experimental hardware

**Fig. 2:**
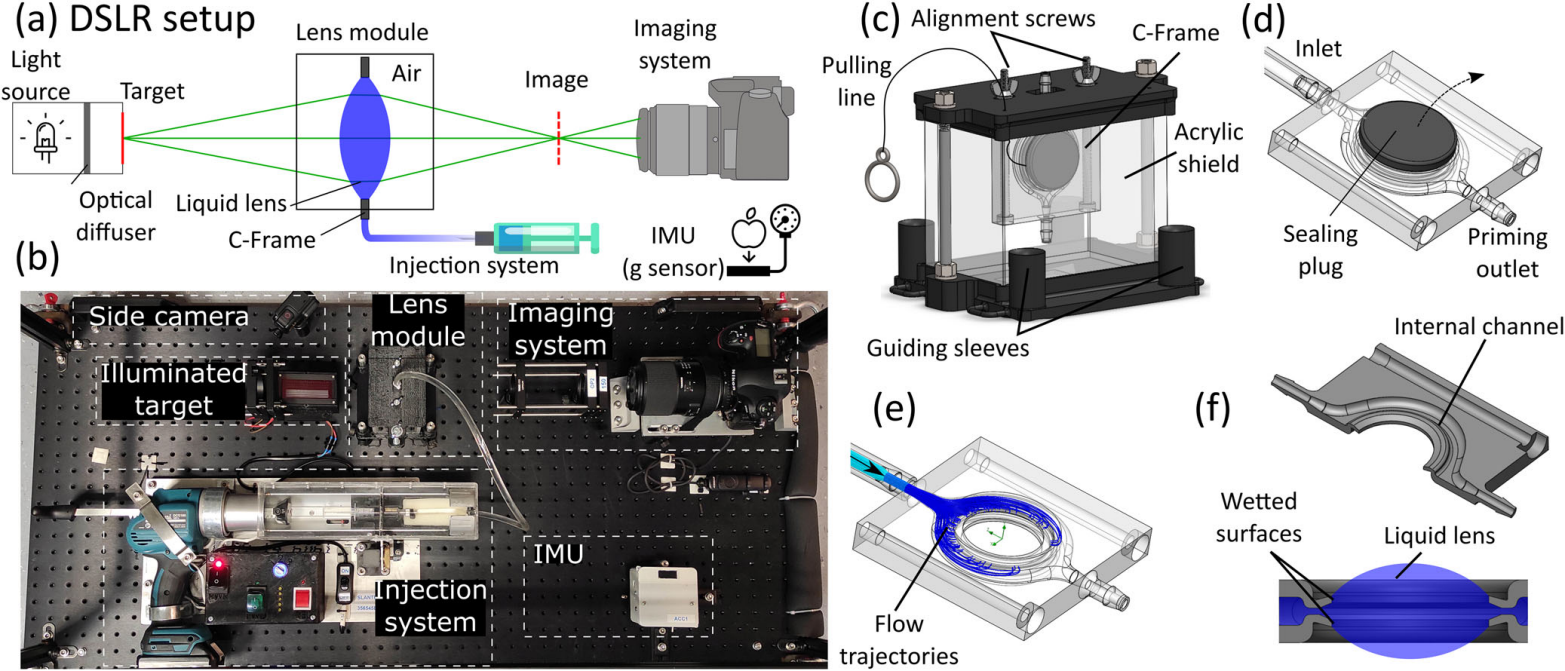
The experimental setup hardware. **a** Schematic and **b** photo of the experimental hardware, showing the central elements. In microgravity, the lens is formed by injecting liquid into the C-Frame contained within the aquarium module. In parallel, an optical system characterizes the resulting lens by imaging through it, a side camera captures a video of the process, and a g-sensor records the proper acceleration. The entire system is mounted on an optical breadboard for structural and optical robustness. **c** The lens module, showing the C-Frame enclosed in the acrylic shield with two screws used for alignment. The module is aligned by sliding its guiding sleeves along four vertical rods on the breadboard, and the pulling line is used to pull the plug before injecting the liquid through the primed channel. **d** The hole at the center of the frame is initially blocked by a plug that prevents the liquid in the channel from leaking out in gravity. **e** In microgravity, after pulling the plug and activating the injection system, liquid flows through the channel and subsequently fills the hole. **f** Cross-section of the C-Frame, showing the inner channel and the wetted surfaces which serve as the bounding frame for the resulting liquid lens.

The experiment was executed on a reduced-gravity aircraft (Boeing 727–227 F, Zero-G Corporation, FL, USA) performing parabolic maneuvers, each providing a microgravity time window of roughly 15 s. Figure 2a shows a general schematic of the hardware structure. Liquid is pushed into a bounding frame to create the lens under test (LUT). A g-sensor (accelerometer) registers the proper acceleration (i.e., the acceleration relative to free-fall), and a side camera (GoPro Hero 7 Black) video records the entire process. Two types of experimental setups were used, which differ only in the way that the LUT was characterized in-situ—one by capturing an image of a test target through the LUT using a DSLR camera (‘DSLR setup’, shown in Fig. 2a), and the other by measuring the effect of the lens on incident light using a Shack-Hartmann Wavefront Sensor (‘SHWS setup’, not shown in the figure).

Figure 2b shows a top-view photo of one of the DSLR setup. A syringe was used to inject the optical liquid—silicone oil with several kinematic viscosities: 200, 1000 (Siliconesandmore.com, The Netherlands) and 5000 cSt (Dow Inc., USA)—into a bounding frame enclosed within an acrylic shield. Those liquids and viscosity values were selected since they are commercially available, easy to handle and cover the relevant working range for this application. We automated the injection using a customized, high-torque pump based on an electrical caulking gun powered by an 18 v Li-ion battery (Makita DCG180). We controlled the DC motor of the gun directly with a microcontroller (Arduino Mega 2560) and an H-bridge driver (HIP4081AIVZ, Shenzhen LC Technology co.). We configured the injection system to accurately inject the required amount of viscous liquid within the first few seconds of each microgravity maneuver. The injection system worked in open loop, based on pre-calibration of the flow rate for each viscosity as a function of the pulsed width modulation (PWM) signal, to result in a flow rate of about 2 ml s^−1^ for all viscosities. We measured the proper acceleration of the setup using an inertial measurement unit (IMU, SparkFun MPU-6050), interfaced by a microcontroller (Arduino Nano) powered by a set of AA batteries.

Figure 2c–f shows the heart of each setup—the ‘C-Frame’, which is a 3D printed component with a 25 mm diameter hole at its center, serving as the bounding frame. The perimeter of the hole is an internal circumferential channel with a C-shaped cross-section, whose opening is towards the center of the hole. The geometry of the C-Frame was optimized to allow the rapid injection of the required liquid volume within the short microgravity timeframe, with the largest aperture possible in those conditions. The channel’s inlet is connected via a 1.5 mm thick polyurethane tube (rated to a working pressure of up to 10 bar) to a syringe filled with the optical liquid. On the ground, before the experiment, we first sealed the C-Frame hole with a rubber plug and primed the channel by running liquid through it while pushing all the air through the outlet. Once all the air was removed and no bubbles were visible, we sealed the outlet with a clamp. To deploy the lens during the experiment in microgravity, we first pulled the sealing plug out of the hole. We then manually turned on the pump to push liquid from the syringe through the inlet. The liquid flowed radially into the hole until all liquid fronts met and a continuous volume of liquid was formed, creating a bi-convex spherical liquid lens. In the remaining microgravity time (typically 10 s) the formed lens is measured by the imaging system. Upon return to gravity, the liquid lens collapsed to the bottom of the acrylic shield.

Each such lens module (C-Frame within acrylic shield) was used a single time. Between sets of parabolas, the used modules were removed, and new ones were installed to repeat the experiment with a clean assembly. The C-Frame was connected to the module using two alignment screws that allowed lateral displacement for optical alignment during pre-flight assembly. The guiding sleeves at the bottom of each module allowed quick and easy mounting on top of the breadboard platform during the flight, while maintaining the optical alignment. The lens module was positioned such that the optical axis of the liquid lens was parallel to the longitudinal axis of the aircraft.

**Fig. 3:**
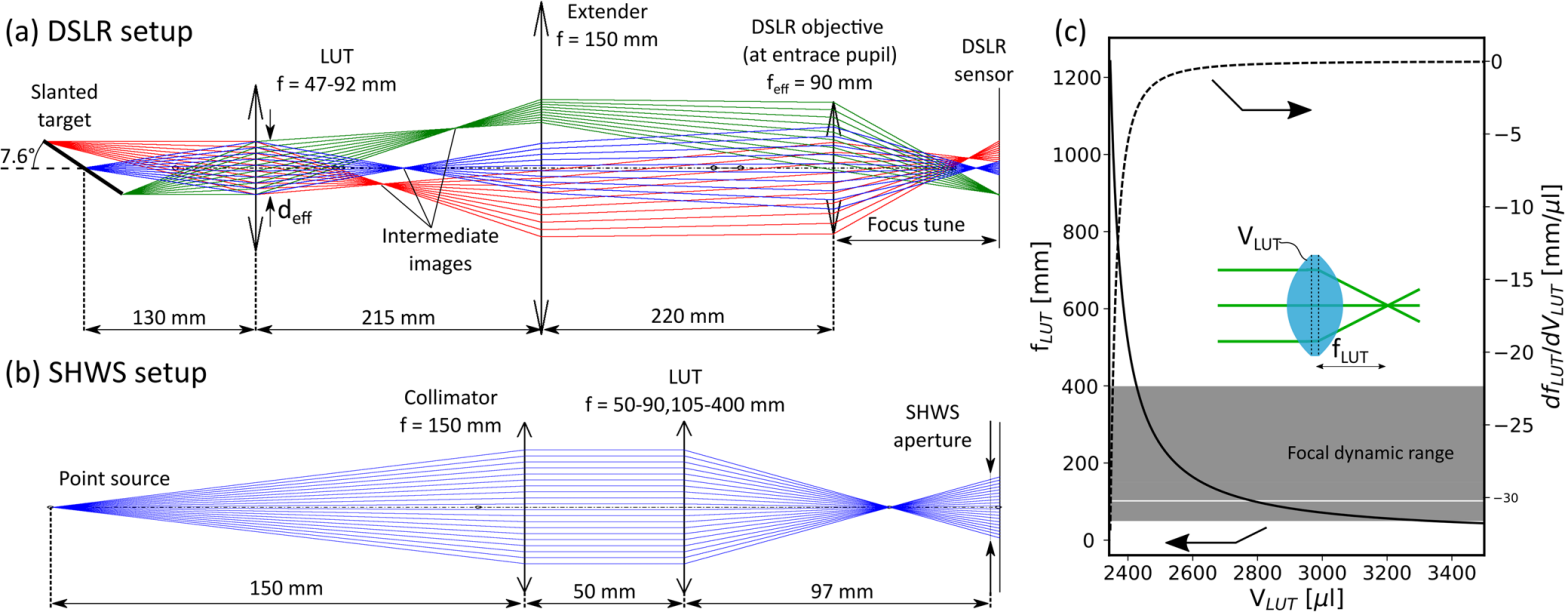
The optical design of the two imaging system types, illustrated by paraxial ray tracing. **a** The DSLR setup design, where a resolution target is imaged through the lens under test (LUT) to form an intermediate real image, which is then magnified by the extender and imaged through the objective to form the final image on the DSLR sensor. Focus is tuned by translating the objective along the optical axis. The target is mounted with a slant angle of 7.6° to add field depth and further extend the focal dynamic range (FDR) of the setup. Ray colors correspond to different field points on the target. **b** The SHWS design, where a point source is collimated to input a plane wavefront to the LUT. The output wavefront is then measured by a Shack-Hartmann wavefront sensor (SHWS). The figure presents the case where the focal length of the LUT is shorter than the distance between the LUT and SHWS plane. When the focal point is close to the sensor, the resolution is insufficient of measurement causing a ‘blind’ region. For larger focal lengths (forming a converging beam at the sensor plane), the wavefront is again resolvable. **c** The focal length of a bi-convex spherical lens with a diameter of 25 mm and refractive index of 1.403, as a function of its volume. The first derivative of this curve is also shown to emphasize the sensitivity of the focal length to changes in the volume, which decrease substantially as the volume increases. The gray area marks the unified focal dynamic range covered by the setups. The horizontal white strip represents the small range of focal lengths that cannot be resolved by the SHWS.

Figure 3a, b shows the optical designs used for the DSLR and SHWS imaging systems, respectively. In the DSLR design shown in (a), the illuminated target is a transmissive resolution test-chart made of a negative chrome mask etched on a glass substrate. The target is back-illuminated by a 530 nm LED board (CREE C503-GCN) with two layers of parchment paper acting as a diffuser, creating an extended source with a highly uniform radiance. The target is imaged through the LUT and creates an intermediate real image that is then magnified by an extender lens (Thorlabs LB1374-A, f = 150 mm, d = 50 mm) and imaged through the objective (Tamron 90 mm f/2.8 with a 12 mm macro extension tube) to create the final image on the DSLR sensor (Nikon D850). To allow greater flexibility in finding the focus during the experiment, the target surface was fixed at an angle of 7.6° from the optical axis and the objective was manually moved against the sensor in real-time. The optical train was designed such that the resulting aberrations could be primarily attributed to the LUT, as shown in Supplementary Fig. 1 in the Supplementary Information (SI).

In the SHWS design shown in (b), a point source (Thorlabs M530F2 530 nm fiber-coupled LED) is positioned at the back focal plane of a collimator lens (Thorlabs LBF254–150-A) to create a planar wavefront. This beam then passes through the LUT and the resulting aberrated wavefront is measured by a Shack-Hartmann wavefront sensor (SHWS, Thorlabs WFS40-7AR).

Since the nominal LUT diameter is fixed to be that of the hole in the C-Frame (25 mm) and the refractive index of the silicone oil is provided by the manufacturer (1.403), uncertainties in volume can be directly translated to variations in the focal length. Figure 3c shows the relation between the volume of the LUT (modeled as two positive spherical caps and a disk) and its focal length, computed using the lens makers’ equation for thick lenses^27,28^. The derivative of this curve is also shown, indicating the sensitivity of the focal length to the volume at different regions.

In both designs, the optical elements, and the distances between them dictate the focal dynamic range (FDR)—the range of values for the focal length of the LUT that can be measured. In the case of the slanted target, at least one point on the slanted target must be focused on the sensor within the travel range of the objective. In the case of the SHWS setup, the wavefront slope must stay within a certain range to ensure that the spot centroids fall within the bounds of their corresponding pixel bins^29,30^, and the entire beam size must be sufficiently large at the sensor plane to capture it with sufficient resolution. Furthermore, due to the limited numerical aperture of the measurement system, the focal length of the LUT dictates its measurable aperture. To accommodate for the uncertainty in focal length, and since changes to the optical system are not feasible during the short microgravity period, the system must be designed to capture a sufficiently large FDR. This poses a tradeoff as the increase in FDR decreases the active aperture.

In both optical trains we optimized the design to extend the FDR as far as possible without excessively reducing the measured aperture of the LUT. Our final design allowed for an FDR of 47–92 mm with about 30% aperture coverage for the DSLR system, and FDRs of 50–90 mm and 105–400 mm with about 50% aperture coverage for the SHWS system. Both the DSLR and SHWS were configured to work continuously, resulting in frame rates in the range of 5–10 fps.

## Results and discussion

**Fig. 4:**
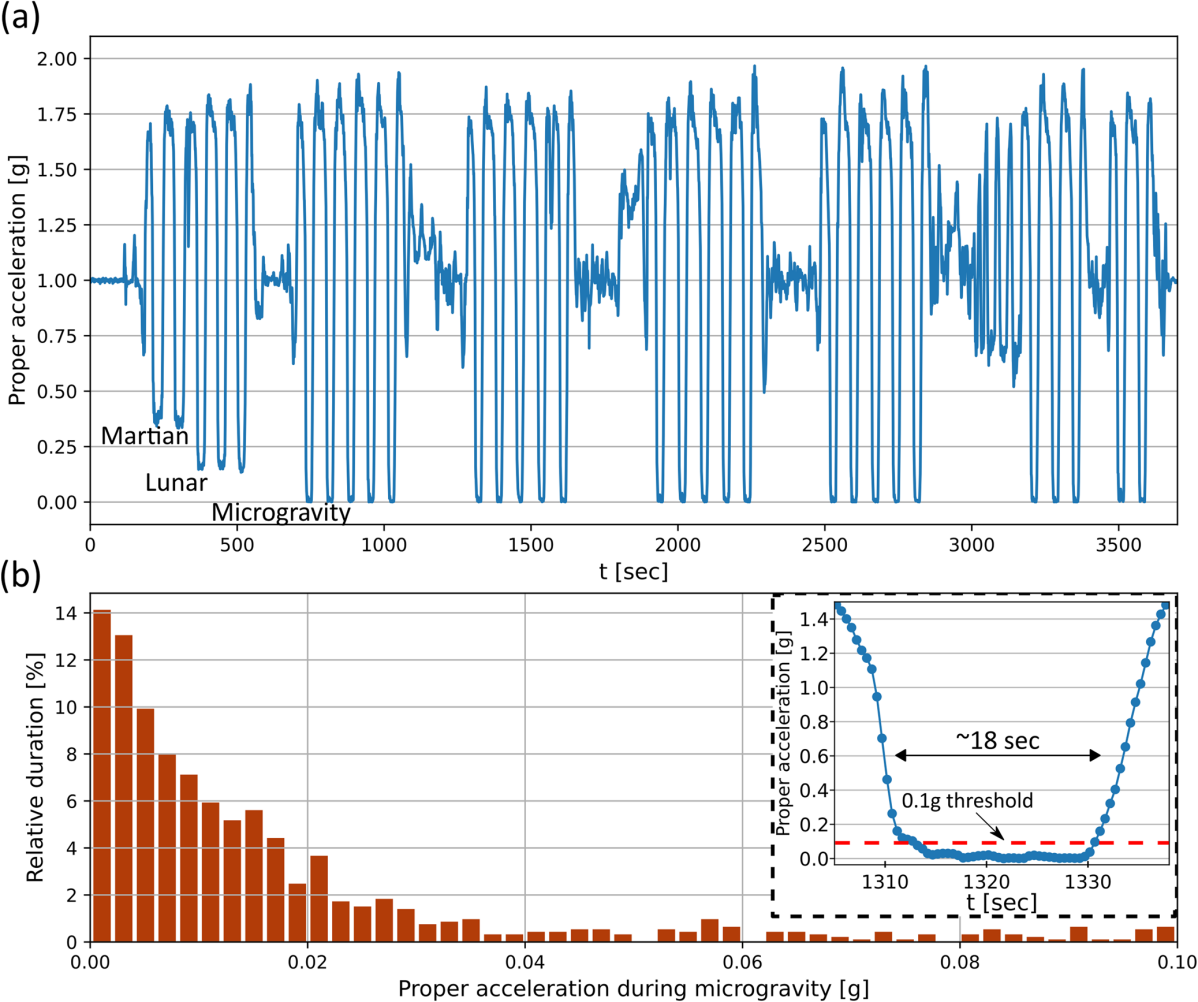
Acceleration profile during the parabolic flight. **a** The proper acceleration magnitude during the flight as measured by the average of two accelerometers located on the experimental setups. The flight profile is composed of one set of reduced gravity conditions (Martian and Lunar), followed by five sets of microgravity. **b** A histogram showing the relative duration of acceleration magnitude during the microgravity periods (which we ad hoc define as g ≤ 0.1). Over 90% of the microgravity time measures below 0.04 g. The inset shows a magnified view of a representative parabolic maneuver in which microgravity conditions were maintained for of 18 s.

Figure 4a shows the proper acceleration data recorded by the accelerometer during the flight, with a sampling frequency of 2 Hz. The planned g profile is easily observed—six sets of five parabolas each. The first set was comprised of two ‘Martian’ (~0.38 g) and three ‘Lunar’ (~0.16 g) maneuvers, while the rest were microgravity maneuvers. To analyze the stability of the microgravity environment provided on the aircraft, we ad hoc define 0.1 g as a threshold value under which we consider the conditions to be ‘microgravity’. Each microgravity parabola produced a single continuous microgravity phase in which this threshold was not exceeded. The inset in Fig. 4b shows a magnified period of one of the maneuvers. Figure 4b presents a histogram showing the relative duration of g values for the union of all microgravity phases in the flight. The histogram shows that during more than 90% of the microgravity time, a clean environment of less than 0.04 g is obtained.

**Fig. 5:**
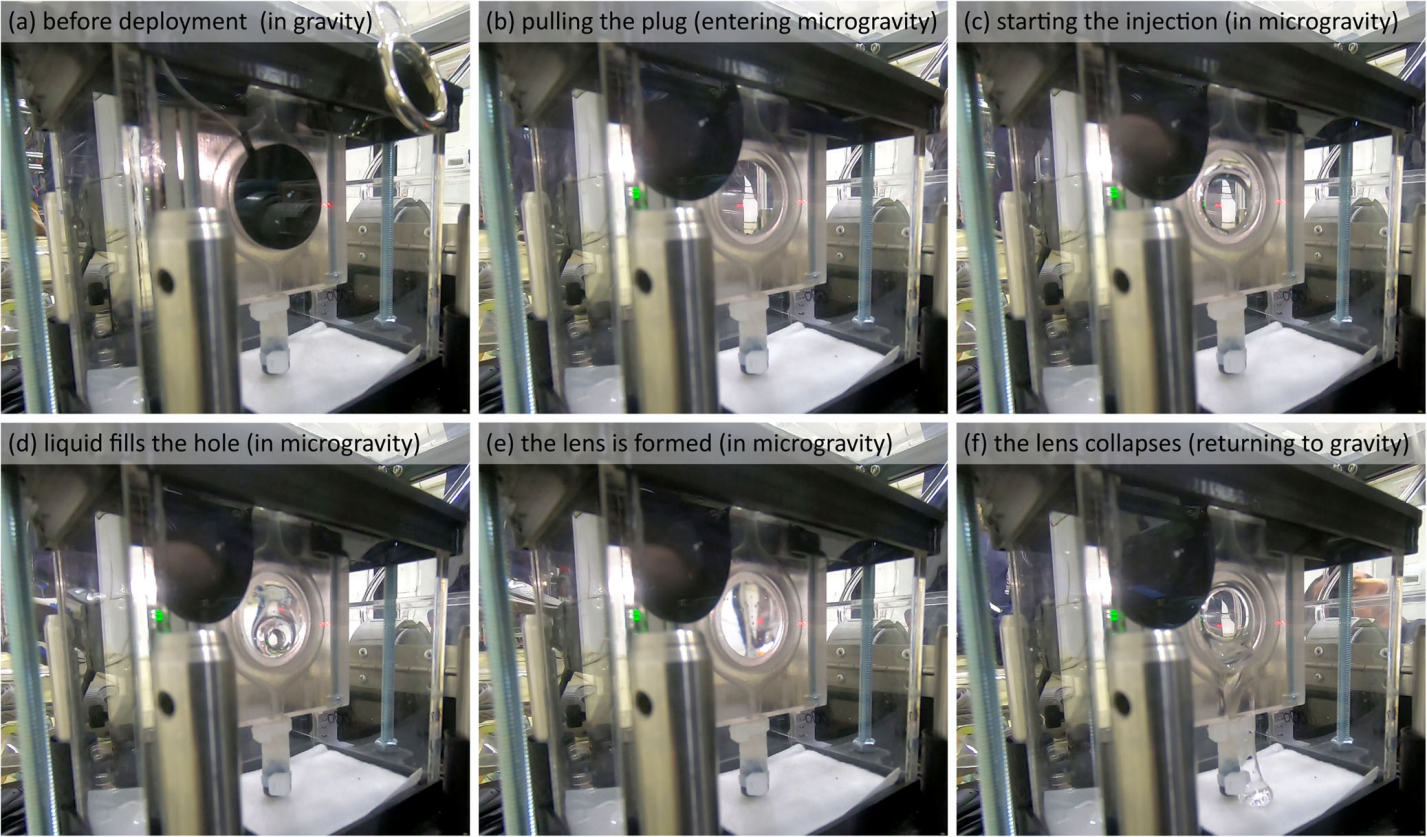
The deployment of a liquid lens in microgravity, as captured by the side camera. **a** Before deployment, the rubber plug seals the hole of the C-Frame. **b** Upon entering microgravity, the rubber plug is pulled, and the injection is initiated. **c**, **d** Liquid flows radially out of the channel and fills the hole until the liquid fronts meet and **e** a liquid lens is formed and is maintained throughout the microgravity duration. **f** Upon returning to gravity, the lens collapses into a puddle at the bottom of the module.

Figures 5a–f show several frames from the side camera during the deployment process (see the SI for a video showing such a deployment). Each experiment started with a new lens module containing a pre-filled C-Frame, with black rubber plug sealing its hole (Fig. 5a). Upon entering microgravity, we pulled the plug out of the C-Frame hole (Fig. 5b) and immediately turned on the pump to initiate the injection of liquid into the frame. In the absence of gravity, the liquid advanced radially from the outer edge of the hole toward its center (Fig. 5c, d). When all the liquid fronts met, a continuous liquid lens was formed (Fig. 5e). When returning to gravity at the end of each parabola, the liquid lens is drained from the lens hole toward the bottom of the acrylic shield and onto an absorbing pad (Fig. 5f).

SI Table 1 provides a table listing all the of the parabolas executed on two flights performed on two consecutive days. On the first flight, out of 14 attempts, 11 resulted in a fully closed lens, as captured by the side camera observing the lens module. Of these, only two were successfully measured (both on the SHWS setup). Between the flight dates, we relaxed the DSLR setup by translating both the LUT and the target 225 mm toward the camera. This results in a wider range of LUT focal lengths that can be captured, at the expense of a smaller measured aperture. The design presented in Fig. 3 corresponds to this modified configuration. On the second flight, 12 out of 15 attempts were successful, of which 8 were measured (five by SHWS and three by the DSLR setup). As can be seen in the SI video, even after the lens is fully formed, the shape of the lens fluctuates, either due to injection dynamics that have not fully settled or due to disturbances to the microgravity environment. In some cases, these changes were too rapid or too significant in amplitude to obtain useful measurements. Also, measurements that have been obtained vary significantly due to those changes—typically at a higher rate than the sampling frequency. In the design of the experiment, we made a conscious decision to favor yield over measurement statistics, and hence created the lenses using liquids of different viscosities and different injected volumes. There is therefore no clear metric that can be used to compare them, and the sample size is too small to provide valuable statistics. The results we report in the following sections are representative of the better lenses captured in the experiments.

## Direct imaging of a resolution test-chart

**Fig. 6:**
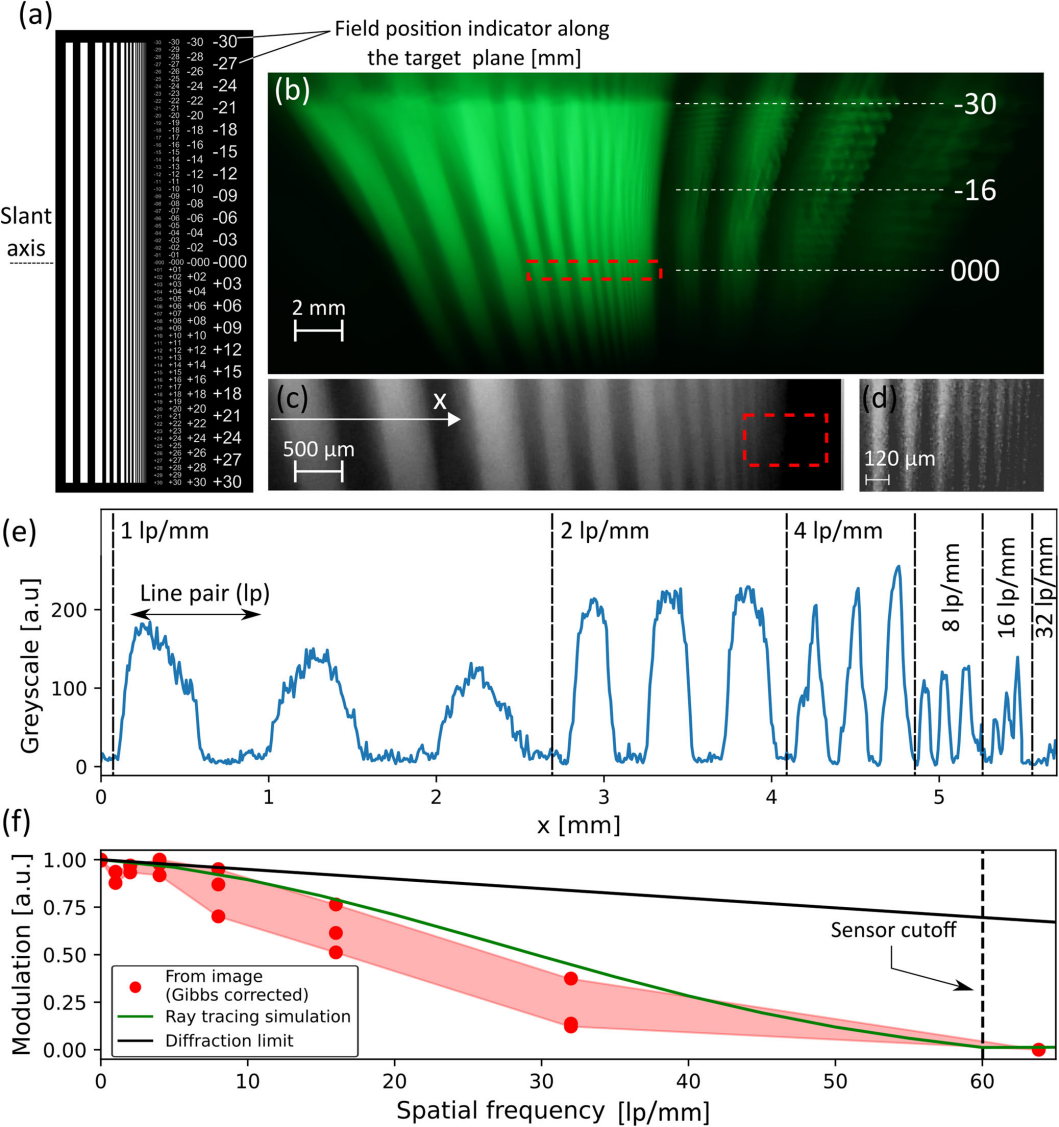
Evaluation of lens performance through imaging of a slanted resolution-target. **a** The slanted target design containing a vertical line pattern with a variable pitch. The target is slanted around the horizontal axis as marked by the dashed line. The numbers to the right denote position along the target plane, (in mm) and are used to indicate the field position (in mm) of each point in the acquired images. **b**–**d** An image and two sequential zoomed-in views (marked by red dashed lines) of the slanted target, taken through the LUT in microgravity. **e** The intensity profile taken along the indicated x-axis, and the associated line-pair groups. **f** Comparison of MTF results obtained from analysis of the target image, from ray-tracing simulation of a lens with the same nominal geometry, and from a diffraction limit calculation. The red region corresponds to the range of values that can be extracted from the target image.

Figure 6a shows the target that consists of pairs of bright and dark strips (line-space pattern). Each line and its adjacent space are of equal width and constitute a line-pair (lp). Three consecutive pairs with the same width form a group. From left the right, the mask is designed to have 10 groups (with three pairs in each) of decreasing line width from 1 mm to 2 μm. The entire target is slanted at an angle around the horizonal axis, allowing a focused image to be obtained for a wide range of focal lengths of the LUT. Figure 6b–d show the target image as acquired by the DSLR setup, and two consecutive zoomed-in sections of the image presented in grayscale. Figure 6e presents the intensity profile corresponding to Fig. 6c, and the resolvable pair groups used for the analysis. For each line-pair group of line width *δ*, we denote the spatial frequency as $\nu =\frac{1}{2\delta }$, and define the directly measured contrast, *C*, as^31^:

5 $$C\left(\nu \right)=\frac{{I}_{{\rm{max }}}(\nu )-{I}_{{\rm{min }}}(\nu )}{{I}_{{\rm{max }}}(\nu )+{I}_{{\rm{min }}}(\nu )}$$

where ${I}_{{\rm{max }}},{I}_{{\rm{min }}}$ are the max and min grayscale values at spatial frequency ν. In our case, the line pattern is vertical, meaning that we only measure spatial frequencies in the perpendicular direction, i.e., on the sagittal plane of the LUT. Since in our case the target image is binary (sharp steps between 0% and 100% transparency), the directly measured contrast is the square wave modulation, also called the contrast transfer function (CTF). The intrinsic modulation transfer function (MTF) is defined only for spatially monochromatic sine wave targets, but can be obtained directly from the CTF by correcting for the Gibbs phenomena at the edges^32^,

6 $$M\left(\nu \right)=\frac{\pi }{4}\left[C\left(\nu \right)+\frac{C(3\nu )}{3}-\frac{C(5\nu )}{5}+\frac{C(7\nu )}{7}-\ldots \right]$$

Since each group consists of three line-pairs, we calculate the CTF and MTF three times for each spatial frequency. Since the Gibbs correction in Eq. (6) requires interpolating the CTF to estimate it in all spatial frequencies, the calculation can be repeated with many permutations of the measured data points. We therefore calculate the MTF separately for the maximum, minimum and intermediate CTF values of each group, leaving a range of values for the MTF at each spatial frequency. Figure 6f presents the resulting MTF range, indicated by the red area. Overall, the MTF shows a gradual decrease in modulation with increasing frequencies, reaching 10% between 40 and 50 lp mm^−1^ (2.3–2.8 cycles mrad^−1^ based on the estimated focal length of the LUT as described below). The sensor cutoff is at the 60 lp mm^−1^, corresponding to half the sensor’s Nyquist frequency, with a pixel size of 4.35 μm. However, we formally indicate the zero modulation at a slightly higher spatial frequency of 64 lp mm^−1^ according to our measured pattern.

To compare the experimental results with optical simulation predictions, we must first estimate the focal length and aperture of the LUT. We achieve this using a ray tracing simulation where those parameters are adjusted until the measured magnification is obtained at the correct field position (identified by the image position along the target plane). At the center of the target (on the optical axis, field position 0), this yields a focal length of ${f}_{{\rm{LUT}}}=57.1\,{\mathrm{mm}}$ (corresponding to an injected volume of 3.2 ml) and an aperture of ${d}_{{\rm{LUT}}}=10.5\,{\mathrm{mm}}$, which fall into their acceptable ranges according to the design. We estimate the uncertainty in the focal length by repeating the calculation with points at the edges of the apparent depth of field ($\pm 10\,{\mathrm{mm}}$ around the optical axis) in Fig. 6b, yielding deviation of $+1\,{\mathrm{mm}}$ and $-2.8\,{\mathrm{mm}}$ from the nominal focal length.

The green line in Fig. 6f presents the simulated MTF of the same optical train, where the LUT is assumed to be a perfectly spherical bi-convex lens with the same focal length and aperture as the measured LUT. This serves as a reference since a spherical lens is the theoretical shape that can be obtained by fluidic shaping in microgravity^25^. We can see that generally, the measured MTF curve agrees with the ray tracing solution. In most spatial frequencies, the experimental results are below those of the simulation, which can be associated with deformations of the liquid surfaces that introduce aberrations. Interestingly, the ultimate resolution limit (i.e., the intersection point of the MTF curve with the horizontal axis) is almost the same for both the simulation and LUT. This clearly shows that the LUT is indeed an optical lens that can function within an optical system. For completeness, we provide the MTF of a diffraction limited lens with the same focal length and aperture. As observed, the MTF of even the ideal spherical lens is far from the diffraction limited one, due the inherent spherical aberration.

## Shack-Hartmann wavefront sensing

**Fig. 7:**
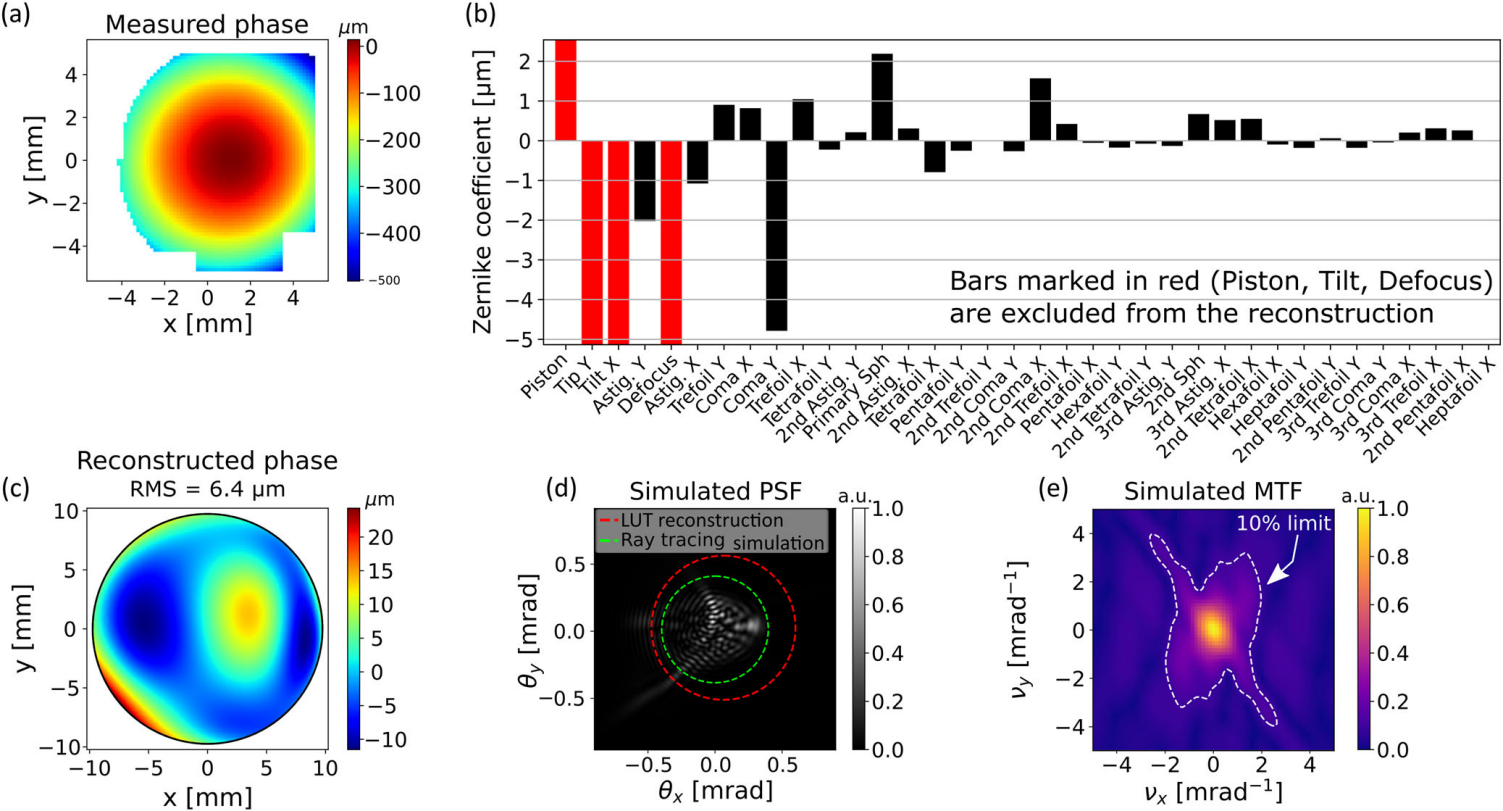
Evaluation of lens performance through wavefront sensing. **a** The raw data, as obtained by the SHWS, showing the measured phase map at the sensor plane. White pixels correspond to points at which the SHWS could not determine the wavefront slope. **b** The Zernike coefficients of the phase map. The scale is adjusted according to the magnitude of all coefficients except for Piston, Tilt and Defocus (marked in red), as they are excluded from the phase reconstruction. **c** The phase at the sensor pupil plane, as reconstructed from the Zernike coefficients in after removing Piston, Tip, Tilt and Defocus (marked in red in ‘**b**’), showing the resulting RMS and P-V of the bare aberrated wavefront. **d** Simulated PSF of the reconstructed phase, after propagating it to the focal plane of the LUT. The red and green circles show the 83.8% encircled energy for the LUT and for an equivalent spherical lens (via ray tracing). **e** Simulated MTF of the reconstructed phase.

Figure 7 presents the wavefront sensing analysis for one of the liquid lenses successfully captured in microgravity. Figure 7a shows the raw measured phase map as it was recorded on the sensor. Figure 7b shows the Zernike decomposition of this phase map, where each vertical bar represents the amplitude of the corresponding Zernike mode coefficient. To estimate the aperture and focal length of the LUT we first calculate the radius of curvature of the wavefront using the Defocus term (with a Zernike coefficient of ${Z}_{4}=-131.1\,\upmu {\mathrm{m}}$)^33,34^. We then find the paraxial focal length and the aperture of the LUT by adjusting these parameters in a ray tracing simulation until the measured wavefront radius of curvature is obtained. This yields an estimated focal length of ${f}_{{\rm{LUT}}}=61.2$ (corresponding to an injected volume of 3.1 ml) and a measured aperture of ${d}_{{\rm{LUT}}}=19.5\,{\mathrm{mm}}$. To estimate the uncertainty in the focal length, we calculate the sensitivity of the residual error (${\chi }^{2}$) in the Zernike fit to changes in the fitted parameter ${Z}_{4}$ (defocus), based on the typical wavefront measurement error (provided by wavefront sensor manufacturer to be $0.0211\,\upmu {\mathrm{m}}$), according to the procedure for least-square fits, as described in Press et al.^35,36^. This results in a focal length variation of $8.9\,\upmu {\mathrm{m}}$, which is significantly smaller than the geometrical uncertainly related to positioning of elements in the system. We estimate this uncertainty to be 0.1 mm, which is thus also the uncertainly in the focal length. After acquiring the values for the focal length and aperture, we discard Piston, Tip, Tilt and Defocus (marked in red in Fig. 7b) for the rest of the analysis. The main aberration modes observed in Fig. 7b are coma and astigmatism. Since the incoming beam is collimated to within a wavefront error of $\lambda /10$ ($53\,{\mathrm{nm}}$), such asymmetric aberrations suggest corresponding asymmetric deformations on the liquid surfaces, which we suspect to be related to either liquid dynamics from the injection process or non-uniform wetting of the C-Frame surfaces during deployment. Similar deformations can be seen in the SI video, and are common in other deployed lenses as well. Fig. 7c shows the reconstructed phase map by summing all the Zernike terms, excluding Piston, Tip, Tilt and Defocus. The resulting root-mean-squared (RMS) wavefront error is $6.4 \, {{\mu {\text{m}}}}$ and represents actual aberrations caused by the LUT. Ideally, the expected geometry of the LUT is a bi-convex spherical lens, which would result in spherical aberration in the form of a quartic radial function^37^. The expected wavefront error RMS of such a lens can be computed by ray tracing to be 3.1 $\upmu {\mathrm{m}}$ (see Fig. S2 in the SI). The wavefront error phase observed in Fig. 7c is clearly not a quartic function and has a much larger RMS error. We therefore conclude that the reconstructed phase in Fig. 7c is mainly associated with surface irregularities (i.e., ‘figure errors’) of the LUT, which we thus estimate to be on the order of several microns.

Figure 7d, e present two figures-of-merit for assessing the performance of a lens. Fig. 7d shows the simulated point-spread-function (PSF) representing the distribution of intensity that would be obtained at the focal plane if the LUT is used to focus a collimated beam. A convenient way to compare point spread functions, especially for under-corrected optical systems that are far from the diffraction limit, is by drawing the encircled energy around the centroid of the PSF. In a diffraction limited system, the Airy disk contains 83.8% of the total power^27^. We therefore define the size of a PSF by encircling 83.8% of the power, and present this for both the LUT (1.01 mrad in diameter) and an equivalent spherical lens simulated via ray tracing (0.80 mrad in diameter). Figure 7e presents the two-dimensional MTF obtained from the reconstructed phase, showing a reduction to 10% around 2–3 cycles mrad^−1^—consistent with the result obtained using target imaging.

## Conclusions and outlook

In this work, we demonstrated the feasibility of shaping liquids into lenses under microgravity conditions. Our theory predicts under these conditions the formation of a bi-convex spherical lens, and our target imaging and wavefront sensor measurements indicate optical performance comparable with a spherical lens (as measured by the MTF).

One of the main difficulties in parabolic flight experiments is the time ‘budget’, i.e., achieving the desired functionality within each parabolic maneuver. Specifically, in our experiments, the challenge was in injecting a very precise volume of liquid in this short period of time. This precision is needed to produce a lens with a focal length that is within the tolerance of the optical system. Our working point was based on open-loop injection which could not account for any unexpected variations in the system, such as due to temperature and pressure changes in the cabin causing expansion or contraction of the liquid. We therefore designed the optical system to accommodate for such variations—this was achieved by the combination of a reduced aperture and the slanted target, both of which increased the focal dynamic range. This approach was proved to be successful, as we were able to measure 10 out of the 23 deployed lenses, across different liquids with a wide range of viscosities and injected volumes. A future improved experimental system could include a feedback loop based on linear encoders of the injection system, on an online flow rate measurement, on the optical measurement, or on any combination of these. This would allow to increase the measured aperture and to better tailor the measurement for the specific LUT.

There are several potential sources of optical aberrations in the system. Our dynamic measurements clearly showed variations in the shape of the lens surfaces during the microgravity periods. We cannot rule out that the reason for this is deviations from 0 g (either static or dynamic), but since our accelerometer measurements indicate a fairly stable microgravity environment, we believe that the main source of variations is the dynamic response of the liquid to the rapid injection. Another possible source of optical aberrations in a lens are residual bubbles in the tubing or ones that are formed due to gas trapping during injection. However, it is important to note that this is not a fundamental limitation of the method, but merely a result of the strict limitations of parabolic flights. The injection system was designed to rapidly create and measure the lenses during the short microgravity time frame, which constrained our flow rates to be relatively high, leaving no time for filtering the bubbles or removing them after the injection. In a different experimental environment, with longer microgravity duration (e.g., in-orbit), there should be enough time to prevent bubbles from entering the lens in the first place, or remove them after creating the lens.

An important part of our experiments was demonstrating the deployment of a liquid lens. For this goal, the time ‘budget’ is an inherent limitation of parabolic flights and introduces an optimization problem that need to be solved—injecting the liquid sufficiently fast (particularly if seeking to create larger apertures) to leave time for the lens to settle before measurement, while also minimizing the introduction of bubbles. However, if one wishes to focus solely on characterizing a liquid lens under microgravity, one possible approach to minimize liquid oscillations and bubbles would be to skip the deployment process and observe a volume of liquid that nearly fills a horizontal dish and wets its edges, forming a concave lens under microgravity.

Our focus in this work was on lenses, i.e., measuring transparent liquids as refractive elements. Another important category of optics is mirrors, which are useful in space applications due to their spectral range and achromaticity, and lower mass. Thus, in future experiments it would be of interest to consider reflective liquids. An experimental system that is aimed at measuring reflection from a liquid surface would also allow for an easier reconstruction of the surface’s shape. This is because each surface would be measured independently, and the measurement would not be affected by volumetric optical properties.

We envision a set of experiments for maturation of the technology toward applications in space manufacturing and telescopes. The limited duration of microgravity conditions during the parabolic flights was sufficient to demonstrate the concept of fluidic shaping under microgravity, but insufficient to allow curing of liquid polymer lenses, even for the fastest reacting polymers that we are aware of. It is also insufficient for complete relaxation of transient effects. Furthermore, deployment of larger aperture optics is not possible within the space and time constraints of such flights. The immediate next step would be to perform experiments onboard the International Space Station (or other orbital flight), providing a longer period of high-quality microgravity. This would enable curing and solidifying high quality lenses over a larger range of apertures and would effectively extend current in-space additive manufacturing capabilities to optics. Another step would be experiments with reflective liquids, demonstrating that the method could be extended to the creation of mirrors. As seen in our experiment, liquid dynamics play an important role in the resulting optical quality. Thus, special attention should be given to the investigation of liquid-structure dynamics, in the context of their effect on the liquid surface shape and optical performance under in-space conditions.

### Supplementary information

Reporting Summary

Supplementary video

Supplementary information

Experimental log

Raw wavefront sensor data, used for creating Figure 6 in the paper.

A Python script for loading and ploting the wavefront sensor data.

Raw DSLR image, used for creating Figure 5 in the paper.

Complete accelerometer data from December 10th flight.

## Data Availability

The raw data from the accelerometer and the DSLR and SHWS setups on which the figures in the paper are based on are included in the Supplementary Information. The complete raw data is available upon request.

## Code availability

A Python code for reading the SHWS data is included in the Supplementary Information. The code that was used to calculate the Zernike decomposition and estimate the PSF and MTF from the wavefront data is available upon request.
