# Supplementary material for: Fluidic shaping and in-situ measurement of liquid lenses in microgravity
Source: NPJ Microgravity. 2023 Sep 11;9:74. doi: 10.1038/s41526-023-00309-9 (PMC10495324; doi:10.1038/s41526-023-00309-9)
Supplement: Supplementary file 3 — Supplementary information [file 41526_2023_309_MOESM3_ESM.pdf]

Supplementary Information

Fluidic Shaping and *in-situ* Measurement of Liquid Lenses in Microgravity

Omer Luria [a], Mor Elgarisi [a], Valeri Frumkin [a,§], Alexey Razin [a], Jonathan Ericson [a], Khaled Gommed [a],

Daniel Widerker [a], Israel Gabay [a], Ruslan Belikov [b], Jay Bookbinder[b],

Edward Balaban[b,*] and Moran Bercovici [a,*]

[a] Faculty of Mechanical Engineering, Technion – Israel Institute of Technology, Haifa, Israel

[§] Current affiliation: Department of Mathematics, Massachusetts Institute of Technology, Cambridge, MA, USA

[b] NASA Ames Research Center, Moffett Blvd., Moffett Field, CA, USA

[*] Corresponding authors: edward.balaban@nasa.gov (E.B), and mberco@technion.ac.il (M.B)

Additional files provided in the Supplementary Information:

1. **SI Video - Fluidic Shaping in parabolic flights.mp4:** A video showing a lens deployment process.
2. **Experiment log - Fluidic Shaping in parabolic flights.pdf:** The complete list of experiments conducted.
3. **10Dec2021 accelerometers.xlsx:** Complete accelerometer data from December 10th flight.
4. **DSC_2233_00001_(8.33_um_per_pix).tif:** Raw DSLR image, used for creating Figure 5 in the paper.
5. **sub_Wavefront_data.csv:** Raw wavefront sensor data, used for creating Figure 6 in the paper.
6. **Wavefront_reader.py:** A Python script for loading and plotting the wavefront sensor data. Requires numpy (tested with 1.23.2), matplotlib (tested with 3.5.3), and plyer (tested with 2.1.0).

(a)

#	Surface Type		Comment	Radius		Thickness		Material		Coating	Semi-Diameter		Chip Zone	Mech Semi-Dia		Conic	TCE x 1E-6	Focal Length	OPD Mode
0	OBJECT	Standard	Slanted target	Infinity		129.630					2.000		0.000	2.000		0.000	0.000		
1		Standard	LUT front	46.063		4.700		1.40,0.0	M		5.259		0.000	12.500		0.000	0.000		
2		Standard	LUT back	-46.063	P	209.150					5.286		0.000	12.500	U	0.000	0.000		
3	(aper)	Standard	Condenser front	153.280		7.240		N-BK7			25.400	U	0.000	25.400		0.000	-		
4	(aper)	Standard	Condenser back	-153.280	P	220.000					25.400	U	0.000	25.400		0.000	0.000		
5	STOP	Paraxial	DSLR objective			104.336	V				7.000	U	-	-			0.000	90.000	1
6	IMAGE	Standard		Infinity		-					0.977		0.000	0.977		0.000	0.000		

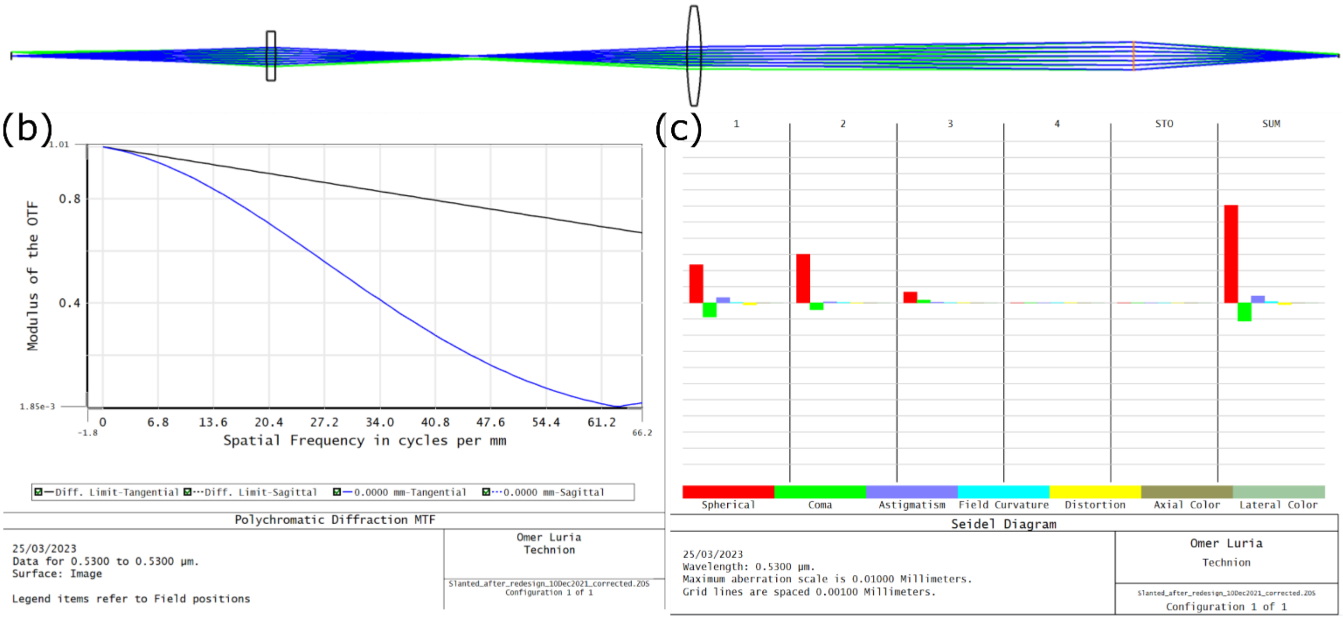

Supplementary Figure 1*: Ray tracing simulation of the DSLR setup in Zemax. (a) The lens data table. The design of the Tamron objective is proprietary and therefore, assuming its resolving ability is far better than that of the LUT, we modeled it as a paraxial surface with a 90 mm focal length. (b) The MTF plot of the optical system, compared to that of a diffraction limited system. (c) The Seidel diagram for the optical setup, showing that the majority of aberrations are associated with the surfaces of the LUT (here, surfaces #1 and #2).*

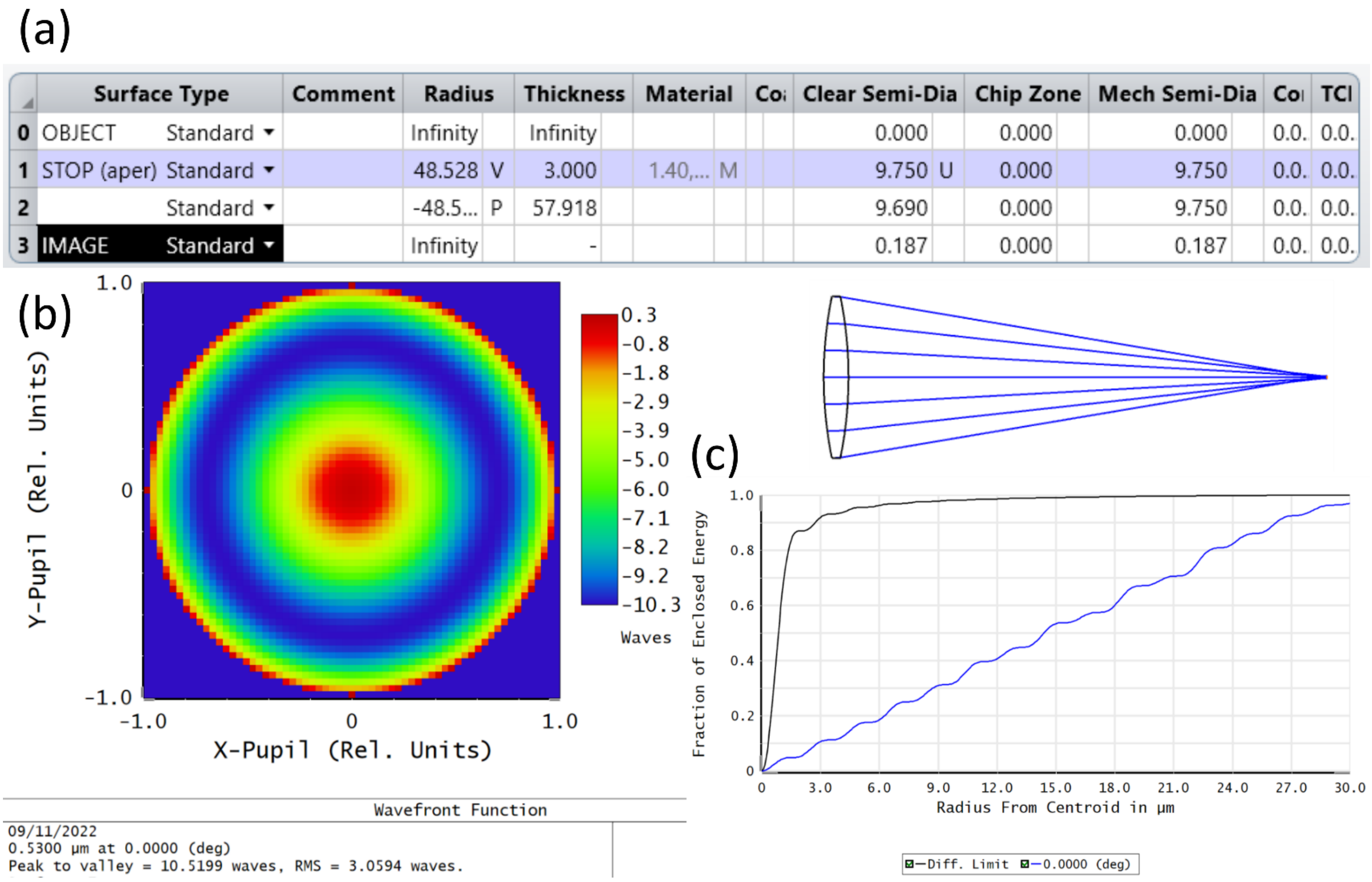

(a)

	Surface Type		Comment	Radius		Thickness	Material		Coa	Clear Semi-Dia		Chip Zone	Mech Semi-Dia	Coi	TCI
0	OBJECT	Standard		Infinity		Infinity				0.000		0.000	0.000	0.0.	0.0.
1	STOP (aper)	Standard		48.528	V	3.000	1.40,...	M		9.750	U	0.000	9.750	0.0.	0.0.
2		Standard		-48.5...	P	57.918				9.690		0.000	9.750	0.0.	0.0.
3	IMAGE	Standard		Infinity		-				0.187		0.000	0.187	0.0.	0.0.

Supplementary Figure 2: *Ray tracing simulation of the SHWS setup. (a) The lens data table. (b) The associated wavefront, after removing pure defocus. (c) The encircled energy for the case of a spherical LUT versus that of a diffraction limited LUT.*
