# Supplementary material for: Fluidic shaping and in-situ measurement of liquid lenses in microgravity
Source: NPJ Microgravity. 2023 Sep 11;9:74. doi: 10.1038/s41526-023-00309-9 (PMC10495324; doi:10.1038/s41526-023-00309-9)
Supplement: Supplementary file 4 — Experimental log [file 41526_2023_309_MOESM4_ESM.pdf]

Flight date	Parabola set #	Parabola #	g profile	Estimated duration [sec]	Active setup	Viscosity [cSt]	Plug success	Injection success	Deployment success	Data success
09-Dec-21	1	1	Martian	17	-	-	-	-	-	-
09-Dec-21	1	2	Martian	17	-	-	-	-	-	-
09-Dec-21	1	3	Lunar	17	Vertical	1000	V	V	V	X
09-Dec-21	1	4	Lunar	17	SHWS	1000	V	V	V	X
09-Dec-21	1	5	Lunar	17	Slanted	1000	V	V	V	X
09-Dec-21	2	6	0g	14	Slanted	1000	V	V	X	X
09-Dec-21	2	7	0g	18	Vertical	1000	V	V	V	X
09-Dec-21	2	8	0g	14	SHWS	1000	V	V	X	X
09-Dec-21	2	9	0g	18	-	-	-	-	-	-
09-Dec-21	2	10	0g	16	-	-	-	-	-	-
09-Dec-21	3	11	0g	16	Vertical	5000	V	V	V	X
09-Dec-21	3	12	0g	14	SHWS	5000	V	V	X	X
09-Dec-21	3	13	0g	14	Slanted	5000	X	X	X	X
09-Dec-21	3	14	0g	12	Slanted	5000	V	V	V	X
09-Dec-21	3	15	0g	14	-	-	-	-	-	-
09-Dec-21	4	16	0g	14	Vertical	5000	V	V	V	X
09-Dec-21	4	17	0g	14	SHWS	5000	V	V	V	V
09-Dec-21	4	18	0g	14	Slanted	5000	V	V	V	X
09-Dec-21	4	19	0g	14	-	-	-	-	-	-
09-Dec-21	4	20	0g	14	-	-	-	-	-	-
09-Dec-21	5	21	0g	15	Slanted	1000	V	V	V	X
09-Dec-21	5	22	0g	16	SHWS	1000	V	V	V	V
09-Dec-21	5	23	0g	14	Vertical	1000	V	V	V	X
09-Dec-21	5	24	0g	14	-	-	-	-	-	-
09-Dec-21	5	25	0g	15	-	-	-	-	-	-
09-Dec-21	6	26	0g	13	Slanted	1000	V	V	V	X
09-Dec-21	6	27	0g	14	SHWS	1000	X	X	X	X
09-Dec-21	6	28	0g	13	Vertical	1000	V	V	V	X
09-Dec-21	6	29	0g	14	-	-	-	-	-	-
09-Dec-21	6	30	0g	14	-	-	-	-	-	-
10-Dec-21	1	1	Martian		-	-	-	-	-	-
10-Dec-21	1	2	Martian		-	-	-	-	-	-
10-Dec-21	1	3	Lunar	18	Vertical	1000	V	V	X	X
10-Dec-21	1	4	Lunar	20	SHWS	1000	V	V	V	V
10-Dec-21	1	5	Lunar	?	Slanted	1000	V	V	X	X
10-Dec-21	2	6	0g	14	Vertical	1000	V	V	V	X
10-Dec-21	2	7	0g	14	SHWS	1000	V	V	V	V
10-Dec-21	2	8	0g	?	Slanted	1000	V	V	V	V
10-Dec-21	2	9	0g	?	-	-	-	-	-	-
10-Dec-21	2	10	0g	?	-	-	-	-	-	-

10-Dec-21	3	11	0g	13	SHWS	1000	V	V	V	V
10-Dec-21	3	12	0g	13	Vertical	1000	V	V	V	X
10-Dec-21	3	13	0g	13	Slanted	1000	V	V	V	V
10-Dec-21	3	14	0g	12	-	-	-	-	-	-
10-Dec-21	3	15	0g	13	-	-	-	-	-	-
10-Dec-21	4	16	0g	13	SHWS	5000	V	V	V	V
10-Dec-21	4	17	0g	14	Vertical	5000	V	V	X	X
10-Dec-21	4	18	0g	13	Slanted	5000	V	V	V	V
10-Dec-21	4	19	0g	18	-	-	-	-	-	-
10-Dec-21	4	20	0g	17	-	-	-	-	-	-
10-Dec-21	5	21	0g	14	SHWS	5000	V	V	V	V
10-Dec-21	5	22	0g	?	Vertical	5000	V	V	V	X
10-Dec-21	5	23	0g	14	Slanted	5000	V	V	V	
10-Dec-21	5	24	0g	15	-	-	-	-	-	-
10-Dec-21	5	25	0g	15	-	-	-	-	-	-
10-Dec-21	6	26	0g	15	SHWS	200	V	V	V	
10-Dec-21	6	27	0g	17	Vertical	200	X	X	X	X
10-Dec-21	6	28	0g	13	Slanted	200				
10-Dec-21	7	29	0g	14	Vertical	200	V	V	X	X
10-Dec-21	7	30	0g	15	-	-	-	-	-	-
